# COVID-19–Related Health Inequalities Induced by the Use of Social Media: Systematic Review

**DOI:** 10.2196/38453

**Published:** 2022-11-15

**Authors:** Yi Shan, Meng Ji, Wenxiu Xie, Xiaomin Zhang, Harrison Ng Chok, Rongying Li, Xiaobo Qian, Kam-Yiu Lam, Chi-Yin Chow, Tianyong Hao

**Affiliations:** 1 Nantong University Nantong China; 2 University of Sydney Sydney Australia; 3 City University of Hong Kong Hong Kong China; 4 Macquarie University Sydney Australia; 5 Western Sydney Local Health District Parramatta Australia; 6 South China Normal University Guangzhou China

**Keywords:** systematic review, social media use, health inequalities, COVID-19, mobile phone

## Abstract

**Background:**

COVID-19–related health inequalities were reported in some studies, showing the failure in public health and communication. Studies investigating the contexts and causes of these inequalities pointed to the contribution of communication inequality or poor health literacy and information access to engagement with health care services. However, no study exclusively dealt with health inequalities induced by the use of social media during COVID-19.

**Objective:**

This review aimed to identify and summarize COVID-19–related health inequalities induced by the use of social media and the associated contributing factors and to characterize the relationship between the use of social media and health disparities during the COVID-19 pandemic.

**Methods:**

A systematic review was conducted on this topic in light of the protocol of the PRISMA (Preferred Reporting Items for Systematic Reviews and Meta-Analyses) 2020 statement. Keyword searches were performed to collect papers relevant to this topic in multiple databases: PubMed (which includes MEDLINE [Ovid] and other subdatabases), ProQuest (which includes APA PsycINFO, Biological Science Collection, and others), ACM Digital Library, and Web of Science, without any year restriction. Of the 670 retrieved publications, 10 were initially selected based on the predefined selection criteria. These 10 articles were then subjected to quality analysis before being analyzed in the final synthesis and discussion.

**Results:**

Of the 10 articles, 1 was further removed for not meeting the quality assessment criteria. Finally, 9 articles were found to be eligible and selected for this review. We derived the characteristics of these studies in terms of publication years, journals, study locations, locations of study participants, study design, sample size, participant characteristics, and potential risk of bias, and the main results of these studies in terms of the types of social media, social media use–induced health inequalities, associated factors, and proposed resolutions. On the basis of the thematic synthesis of these extracted data, we derived 4 analytic themes, namely health information inaccessibility–induced health inequalities and proposed resolutions, misinformation-induced health inequalities and proposed resolutions, disproportionate attention to COVID-19 information and proposed resolutions, and higher odds of social media–induced psychological distress and proposed resolutions.

**Conclusions:**

This paper was the first systematic review on this topic. Our findings highlighted the great value of studying the COVID-19–related health knowledge gap, the digital technology–induced unequal distribution of health information, and the resulting health inequalities, thereby providing empirical evidence for understanding the relationship between social media use and health inequalities in the context of COVID-19 and suggesting practical solutions to such disparities. Researchers, social media, health practitioners, and policy makers can draw on these findings to promote health equality while minimizing social media use–induced health inequalities.

## Introduction

### Background

Currently, the focus of web use has shifted from primarily unidirectional information-seeking to web-based interaction, information sharing, and collaboration [1]. “The increased use of Web 2.0...provides potential opportunities to engage people in health-related issues, stimulate an active role in their health care, connect them with others and evidence-based interventions, and create social action focused on the social determinants of health disparities,” thereby offering underserved and underrepresented populations potential access to essential health information resources and social support for addressing health care issues [2]. *Social media* and *social networking* started being increasingly used to depict the intrinsic characteristics of tools, apps, and functions on Web 2.0 [2]. Compared with traditional media (eg, newspapers, magazines, television, and radio), social media offer easy access to information that can be distributed to larger audiences more rapidly and cost-effectively [3,4]. “The rapid adoption of the Internet and computing technologies by all sectors of modern society has made them an indispensable part of our daily work and life” [5]. Popular social media platforms (eg, Facebook, Twitter, and web-based health community forums) have been applied by health service providers to promote health and facilitate community engagement [6-9]. Social media has been widely and frequently adopted to disseminate information, especially during a crisis or emergency [10]. Ever since the outbreak of COVID-19, diversified social media platforms have been serving as prioritized resorts to publicize COVID-19–related information to the public worldwide owing to the vast number of users [4,10].

Social media can enhance target populations’ access to health services and facilitate information flow and service uptake, but little agreement has been reached on the best practices of social media [8,11] because social media are by no means problem free [10]. The first concern relates to equal access to social media. Given that social media require the use of smart devices, such as smartphones, computers, and laptops, to access the internet, a barrier is imposed on those unable to access these devices. Even among those with such access, the differences in language and computer literacy cause disparities in the quantity and quality of information they receive [5]. Besides, the lack of gatekeeping in social media, the immediate communication of scientific information from discovery to dissemination without calibration, and the public’s nonscientific background have all caused the generation and spread of misinformation, especially during the pandemic [12], posing a great threat to people’s health because preventive and protective practices were compromised by such misinformation.

COVID-19–related health inequalities were reported in a recent study [13], showing the failure in public health and communication. “Health inequality is the generic term used to designate differences, variations, and disparities in the health achievements of individuals and groups” [14], closely associated with social, economic, and environmental disadvantages [15]. Inequalities in health care service access have been well documented [16,17]. Studies investigating the contexts and causes of these disparities pointed to the contribution made by communication inequality or poor health literacy and information access to engagement with health care services [18-20]. However, no study exclusively dealt with health inequalities induced by the use of social media.

### Objective

The objective of this review was two-fold: (1) to identify and summarize COVID-19–related health inequalities induced by the use of social media and the associated contributing factors and (2) to characterize the relationship between the use of social media and health inequalities during the COVID-19 pandemic. This review can thus inform researchers, social media and health practitioners, and policy makers, who can therefore make joint efforts to take full advantage of social media to promote health equality while minimizing social media use–induced health inequalities [21].

## Methods

### Overview

This review was conducted and reported in light of the protocol of the PRISMA (Preferred Reporting Items for Systematic Reviews and Meta-Analyses) 2020 statement [22]. The methods of the review process and the selection criteria were predefined.

### Literature Search

The Medical Subject Headings terms we used for this study were “social media,” “COVID-19,” “SARS-CoV-2,” and “coronavirus.” The keyword search strategy was “(social media [Title/Abstract]) AND (COVID-19* [Title/Abstract] OR SARS-CoV-2 [Title/Abstract] OR coronavirus [Title/Abstract]) AND (equal* [Title/Abstract] OR inequal* [Title/Abstract]).” On March 27, 2022, we conducted keyword searches to retrieve articles concerned with health inequalities induced by social media–related factors in multiple databases: PubMed (which includes MEDLINE [Ovid] and other subdatabases), ProQuest (which includes APA PsycINFO, Biological Science Collection, and others), ACM Digital Library, and Web of Science, without any year restriction. In total, 670 publications were retrieved. Among them, 442, including duplicates, other document types, and non-English papers, were first removed. The articles analyzed and synthesized in this review were selected from the remaining 228 articles based on the predefined selection criteria.

### Selection Criteria

#### Publication Information

No limit was put on the publication year in the keyword searches for relevant literature. No restriction was imposed on the age of the target populations. The selected articles had to be written in English. The articles needed to be research papers published in journals or presented at conferences. Other document types (eg, reviews, abstracts, editorials, workshop summaries, perspectives, opinions, diagnosis methods, and study protocols) were excluded [23]. Studies undertaken in any country were considered.

#### Population

The target population was any group in the public worldwide who experienced social media use–induced health inequalities during the repeated resurgences of COVID-19.

#### Health Inequalities

The health inequalities discussed and summarized in this review could be any aspect related to any health issues, mental or physical. The health inequalities could be experienced anywhere worldwide, so long as they were induced by social media use and related to COVID-19. All studies satisfying these inclusion criteria were selected for the review.

#### Social Media

*Social media* under discussion in this review referred to ways of sharing information, opinions, images, videos, etc, using the internet, especially social networking sites, including WeChat, WhatsApp, Facebook, Twitter, web-based health community forums, etc.

#### Comparator

The comparator could be any form of health inequalities induced by social media. Publications with no comparison were also included because the aim of this review was not to determine the relative degrees of social media–induced health inequalities but to scrutinize the current status of social media–induced health inequalities experienced by people worldwide during the COVID-19 pandemic.

#### Outcomes

The outcomes of the selected studies we considered were as follows: participants’ physical and mental health inequalities induced by the use of social media and the associated contributing factors.

#### Study Design

The designs of eligible studies were quantitative, qualitative, or mixed methods approaches adopted for investigating the outcomes mentioned above. Pilot studies and case studies were included because both types of studies could shed light on the study outcomes above.

### Study Selection

Microsoft Excel was used to manage the retrieved articles and collect data from them. The selection of eligible studies was performed in 3 rounds. In the first round, duplicates, non-English articles, and other document types were all excluded. In the second round, 6 reviewers (YS, XQ, RL, YC, XW, and TS) reviewed titles and abstracts independently against the selection criteria. Any disagreements were settled through discussion among these reviewers and consultation with another 2 reviewers (MJ and WX). In the third round, 2 reviewers (MJ and YS) reviewed the full texts of the remaining articles to further identify eligible studies drawing on the selection criteria. The PRISMA flowchart of the screening and full-text review was produced by WX.

### Quality Assessment

To verify the relevance and methodological solidity of the selected studies, we evaluated the study purpose, literature review, methodology, results obtained, risk of biases in terms of sampling, outcome measures, and conclusions of the selected studies using a modified version of the quality assessment scale adapted from a recent study [23], which was based on the critical review forms of Critical Review Form—Qualitative Studies and Critical Review Form—Quantitative Studies [24,25]. Specifically, 10 questions, presented in Textbox 1, were used to assess the quality of the selected studies. 1 and 0 meant a *yes* answer and a *no* answer to any of the 10 questions, respectively. The maximum quality score for each study was 10. Any study whose quality score was below 6 was excluded from the review.

**Quality assessment scale of the selected studies.**

1. Was the purpose stated clearly?
2. Was relevant literature reviewed?
3. Was the sample described in detail?
4. Was the sample size justified?
5. Were the outcome measures reliable?
6. Was the intervention described in detail?
7. Were results reported in terms of statistical significance?
8. Were the analysis methods appropriate?
9. Was clinical importance reported?
10. Were conclusions appropriate given the study methods and results?

### Data Extraction and Synthesis

Two reviewers (MJ and YS) extracted data from the eligible articles meeting the quality standard by following a standardized form, in which data items included first author’s name and reference, publication year, country, target population, sample size, study design, data collection methods, social media forms, types of health inequalities, social media–related factors for health inequalities, comparator (if applicable), and recommended resolutions.

## Results

### Study Selection

**Figure 1:**
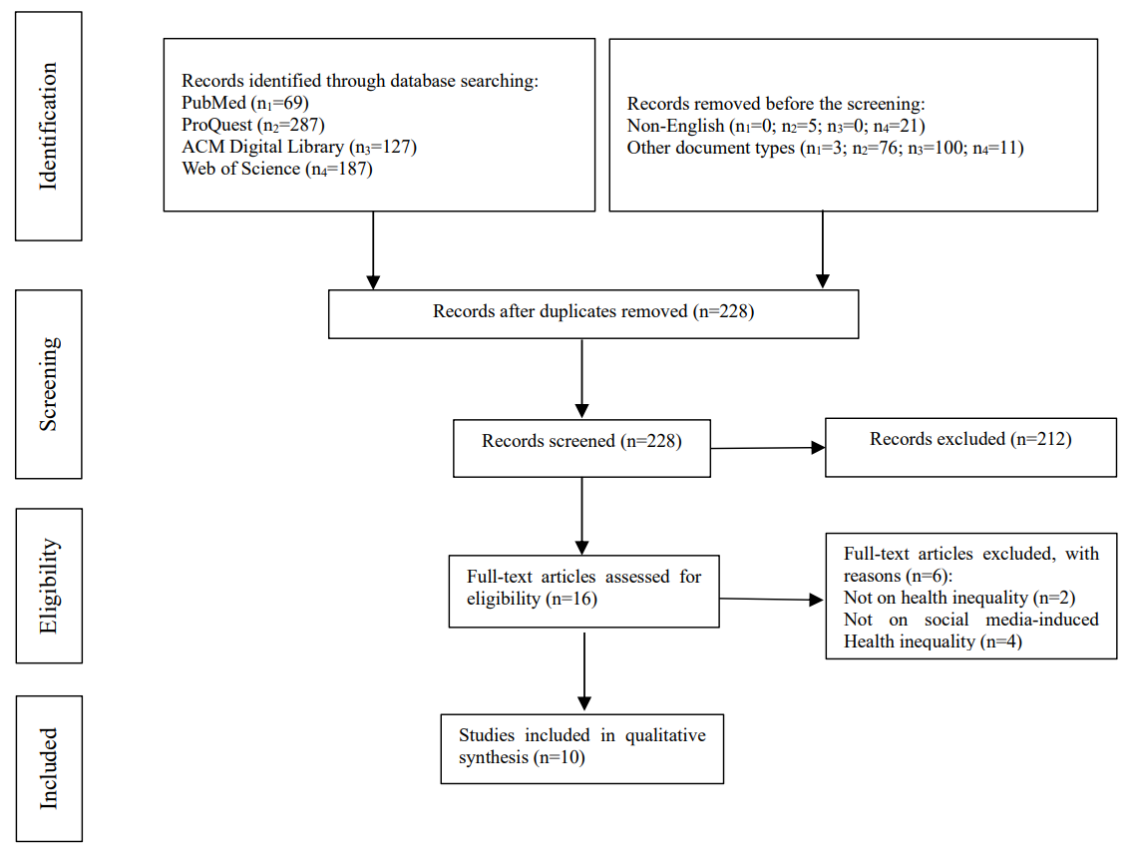
PRISMA (Preferred Reporting Items for Systematic Reviews and Meta-Analyses) flowchart of the selection of eligible studies.

In the first round of selection, 442 articles (including 226 duplicates, 26 non-English articles, and 190 articles of other document types) were removed. In the second round, 212 articles were excluded because of the violation of at least 1 item in the selection criteria. In the third round, 6 articles were removed from the remaining 16 articles because they were not concerned with health inequalities (2/6, 33%) or social media use–induced health inequalities (4/6, 67%). Therefore, 10 studies were found eligible and subjected to quality assessment. The selection flowchart is shown in Figure 1.

### Qualitative Analysis

Table 1 shows that except for the study by Shaw [26], of the 10 included articles, 9 (90%) met the inclusion criteria in this systematic review. So, we finally chose these 9 qualified studies [3,27-34] for qualitative synthesis. The most prominent problem of these 9 selected studies was that they did not report the clinical importance (item 9). Besides, 44% (4/9) of studies failed to describe the interventions in detail (item 6) and report results in terms of statistical significance (item 7). According to Zhou and Parmanto [23], the cutoff score for any studies that were qualified for inclusion in a systematic review was 6 (out of a total score of 10). Although failing to meet some of the quality assessment criteria, the 9 studies [3,27-34] were finally included in the qualitative analysis for this systematic review.

**Table 1:** Quality assessment of eligible studies based on Textbox 1 (N=10).

Study	Items of quality assessment											Score, n (%)
	1	2	3	4	5	6	7	8	9	10		
Wang et al [3], 2021	Y^a^	Y	Y	Y	Y	Y	Y	Y	N^b^	Y	9 (90)	
Dai et al [27], 2021	Y	Y	N	Y	Y	Y	N	Y	N	Y	7 (70)	
Almusawi et al [28], 2021	Y	Y	Y	N	Y	Y	Y	Y	N	Y	8 (80)	
Zeng et al [29], 2020	Y	Y	Y	N	Y	Y	Y	Y	N	Y	9 (90)	
Gallagher et al [30], 2021	Y	Y	N	Y	Y	Y	N	Y	Y	Y	8 (90)	
Blevins et al [31], 2021	Y	Y	N	Y	Y	N	N	Y	N	Y	6 (60)	
Shaw [26], 2020	Y	Y	N	N	N	N	N	N	N	Y	3 (30)	
Wade et al [32], 2021	Y	Y	N	Y	Y	N	N	Y	N	Y	6 (60)	
Ambelu et al [33], 2021	Y	Y	Y	Y	Y	N	Y	Y	N	Y	8 (80)	
Wagner et al [34], 2021	Y	Y	Y	N	Y	N	N	Y	N	Y	6 (60)	

^a^Y: study meets the standard of an item of quality assessment.

^b^N: study does not meet the standard of an item of quality assessment.

### Study Characteristics

#### Publication Years

Of the 9 finally selected papers, 8 (89%) studies [3,27,28,30-34] were published in 2021 and 1 (11%) [29] in 2020.

#### Journals

All the 9 selected articles were published in peer-reviewed journals. Each of the following journals contained 11% (1/9) of selected studies: *BMC Public Health* [3], *Disability & Society* [27], *Research in Developmental Disabilities* [28], *Journal of Medical Internet Research* [29], *Journal of Racial and Ethnic Health Disparities* [32], *Annals of General Psychiatry* [33], and *Health Communication* [34]. Of these, 2 papers [30,31] were retrieved from *Social Media + Society*.

#### Study Locations

Of the 9 studies, 3 (33%) studies were undertaken in China [3,27,29], 3 (33%) in the United States [30-32], 1 (11%) in Kuwait and Saudi Arabia [28], 1 (11%) in Ethiopia [33], and 1 (11%) in Germany [34].

#### Locations of Study Participants

We originally intended to identify specific locations of study participants to reveal the health inequalities potentially existing between different areas reported in the 9 selected studies. However, only 33% (3/9) of these studies mentioned the places where the participants were located: city, countryside, or town [3]; Hubei Province, China [27]; and historically Black colleges and universities [32].

#### Study Design

A total of 67% (6/9) of studies were case studies [3,29-31,33,34] and the other 33% (3/9) were cohort studies [27,28,32]. The studies collected data from participants using web-based cross-sectional surveys and questionnaires [3,28,33], semistructured interviews [27,32,34], a web-based survey (extraction of data from web-based data sets) [29,31], and a mixed methods approach [27,30] (a WeChat ethnography research, participant observation, and semistructured interviews [27]; identifying COVID-19–related content through a keywords-based approach, introducing a measure of sustained amplification, and undertaking a qualitative hand-coding [30]).

#### Sample Size

In the 67% (6/9) of case studies [3,29-31,33,34], the sample sizes were 981, 1215, 1401, 212, 445, 929, and 22. In the 33% (3/9) of cohort studies [27,28,32], the sample sizes were 110, 190, and 21. It should be noted that in 22% (2/9) of these studies, the samples were 1215 tweets from 134 Weibo accounts [29] and 212,445 Twitter tweets from 137,746 unique users [31]. Most of the studies (6/9, 67%) [3,27,29-31,33] satisfied the standard of an appropriate number of participants (between 150 and 200) proposed by Dunbar [35]. The remaining studies (3/9, 33%) [28,32,34] used small sample sizes below this standard.

#### Participant Characteristics

Only 56% (5/9) of studies reported the age of the participants, primarily focusing on those aged >16 years [3], ≤60 years [28], ≥18 years [32], 30 to 34 years [33], and 19 to 80 years [34]. With the information provided in the studies, it was impossible to calculate the average age of the participants, and these 56% (5/9) of studies clearly stated the sex of the participants: male and female. Of the 9 studies, 4 (44%) studies [3,29,31,34] had the public as participants; 2 (%) studies [27,28] chose people with disabilities as informants; the other 3 (33%) studies investigated elites [30], college students [26], and educated people with internet access [33]. Merely 22% (2/9) of studies [30,32] reported the races of the participants.

#### Potential Risk of Bias

Various types of potential risks of bias were identified in the 9 studies. A total of 44% (4/9) of studies had a small sample size [27,28,32,34]. In all, 33% (3/9) of studies [3,29,33] reported an uneven size (Table 2). In addition, 11% (1/9) of studies [30] mentioned the bias of the lack of comparison between different types of social media and 11% (1/9) of studies [31] referred to weighing all edges equally as a potential bias. Moreover, 44% (4/9) of studies [3,28,32,33] reported that participants were either predominantly male or female, so there was sex bias in these studies. Besides, the study by Zeng and Li [29] pointed out another bias: only using descriptive statistics and content analysis and failing to investigate the psychology and behavior of the audience. These characteristics of the 9 selected studies are summarized in Table 2.

**Table 2:** Characteristics of the selected studies (N=9).

Study	Journal	Study location	Location of participants	Study design	Study method	Sample size, n	Participant characteristics	Potential bias
Wang et al [3], 2021	*BMC Public Health*	China	City, countryside, or town	Case study	Cross-sectional web-based survey	981	Male and female; aged >16 years; all levels of education; students, workers, farmers, self-employed, employed in enterprises or institutions, retired, unemployed, and other	Uneven sample composition, which is mainly urban residents, young people, and people with a college education or above
Dai and Hu [27], 2021	*Disability & Society*	China	Hubei Province, China	Cohort study	WeChat ethnography research; participant observation; semistructured interviews	190	People with disabilities	Small sample size
Almusawi et al [28], 2021	*Research in Developmental Disabilities*	Kuwait and Saudi Arabia	—^a^	Cohort study	A cross-sectional electronic survey; questionnaire	110	People with hearing loss and no hearing loss; male and female; aged ≤60 years; unemployed, student, employed (non–health care and health care); primary, middle, high school, diploma, bachelor, and postgraduate	Small sample size
Zeng and Li [29], 2020	*Journal of Medical Internet Research*	China	—	Case study	A survey based on data extraction from Weibo accounts	1215 Weibo tweets from 134 sample accounts	The public	Samples not including county-level administrative regions; only evaluating the government Weibo accounts; only using descriptive statistics and content analysis and failing to investigate the psychology and behavior of the audience
Gallagher et al [30], 2021	*Social Media + Society*	United States	—	Case study	A mixed methods approach	1401	Elites of various demographic populations	The lack of comparison with information crowdsourcing on other platforms like Facebook, Reddit, YouTube, WhatsApp, TikTok, etc
Blevins et al [31], 2021	*Social Media + Society*	United States	—	Case study	A survey based on the COVID-19 Twitter data set	212,445 tweets	137,746 unique users	Weighing all edges equally
Wade et al [32], 2021	*Journal of Racial and Ethnic Health Disparities*	United States	Historically Black Colleges and Universities	Cohort study	In-depth interviews; quantitative surveys	21	Students enrolled during the spring 2020 semester; aged ≥18 years; male and female; Black American, Black foreign born, and White American	Small sample size
Ambelu et al [33], 2021	*Annals of General Psychiatry*	Ethiopia	—	Case study	A web-based cross-sectional survey; questionnaire	929	Educated Ethiopian population having access to the internet; male and female; aged 30-34 years	Only sampling communities who could read and write in English and had internet access; only studying the acute psychological impact and possibly being not generalized to subacute and long-term psychological complications
Wagner and Reifegerste [34], 2021	*Health Communication*	Germany	—	Case study	Semistructured qualitative interviews	22	Aged 19-80 years; male and female; the frequency of interpersonal communication about health topics (low to very high) and the extent of digital media use for interpersonal communication purposes (low to very high)	Small sample size

^a^Not available.

### Main Results of the Selected Studies

#### Overview

The types of social media, social media-use–induced health inequalities, associated factors, and proposed resolutions are presented in Table 3. Through this table, we intended to compare the main research results of the 9 selected studies.

**Table 3:** Types of social media, social media-use–induced health inequalities, social media–related factors for health inequalities, and proposed resolutions to social media-use–induced health inequalities reported in the 9 selected studies.

Study	Types of social media	Social media use–induced health inequalities	Social media–related factors for health inequalities	Proposed resolutions to social media-use–induced health inequalities
Wang et al [3], 2021	Internet	Different levels of health knowledge related to COVID-19 among groups with different education levels; the digital health knowledge gap	The use of traditional media, including newspapers, radio, and television failed to improve knowledge levels; different internet media use: web-based media use expanded the COVID-19 knowledge gap between groups with varying education levels	Improving the pertinence in communication ways and contents; building authoritative scientific knowledge communication platforms; developing internet media literacy and scientific literacy
Dai and Hu [27], 2021	Media coverage on COVID-19 on multiple platforms and avenues, for example, live streaming of government press conferences and reports in digital media	People with disabilities inadequate, accessible information on COVID-19 compared with people with no disabilities	Gaps between policies and practices regarding the digital accessibility infrastructure: The accessible web information for people with disabilities focused little on applicable information to meet the individual needs of people with disabilities during the pandemic; Sign language interpreters are commonly nonexistent in official press conferences and television news on COVID-19; The newly developed website for disseminating information on COVID-19 also lacks accessibility design and remains inaccessible to the communities with hearing or visual disability	A self-initiated and volunteer-driven Disability Support Network; the formulation of comprehensive and inclusive communication strategies for people with disabilities, which fully consider multiple dimensions of information for people with disabilities, including formats, content, and situations; government and public service sectors taking more proactive measures to provide inclusive communications and information in emergencies for people with disabilities
Almusawi et al [28], 2021	Social media	Disparities in the use of health information sources	Participants with hearing loss mainly relied on social media, while the group with no hearing loss relied mainly on official government sources; low health literacy preventing the group with no hearing loss from accessing web-based health information.	Bridging the gap in health literacy for individuals with hearing loss was essential in policy and practice to ensure equal access to health care and universal compliance with health directives at the population level: The use of social media and unstandardized dialectic writing on the web Different modes of disseminating information such as written information and QR codes linking to web-based videos in sign language
Zeng et al [29], 2021	Sina Weibo	Disparities in health information released on Weibo between the eastern region and the central and western regions in China; misinformation on COVID-19 information and prevention and treatment	Governments’ low willingness and ability to use government Weibo accounts; the passives state of the social media operations of public health authorities in China; Centers for Disease Control government Weibo accounts inform the public of the latest developments of the epidemic but fail to respond to public inquiries and a large amount of misinformation during the epidemic promptly.	Governments in the central and western regions learned from similar experiences of neighboring governments; governments maintain their social media activity and update daily information frequently; governments use social media as a channel to release public health information and transmit health information to the public promptly
Gallagher et al [30], 2021	Twitter accounts (known as crowdsourced elites)	Twitter accounts receiving disproportionate attention for COVID-19 content during the pandemic; disparity between sustained and episodic amplification of COVID-19 information	Crowdsourced elites varying across demographics in terms of race, geography, and political alignment; different subpopulations preferentially amplifying elites that are demographically similar to them; different subpopulations crowdsourcing different types of elite accounts, such as journalists, elected officials, and medical professionals, in different proportions	Using the disproportionate voice of crowdsourced COVID-19 elites on the web to equitably promote public health information and mitigate misinformation across the networked public.
Blevins et al [31], 2021	Twitter	Misinformation on COVID-19 information and prevention and treatment	Specific actors and networked communities on Twitter spread false information; key voices amplified COVID-19 misinformation on Twitter during the 2020 worldwide pandemic	As networked societies become more accustomed to relying on information from varying sources on social media outlets and other cyberspaces (even for critical medical knowledge), the implications of how they interpret and apply that information in physical spaces was a significant consideration.
Wade et al [32], 2021	Twitter, YouTube, and Google engine	Misinformation on COVID-19 and associated precautions	Students trust social media sources over government organizations such as the Centers for Disease Control and Prevention and World Health Organization	Universities should consider implementing programs to aid in navigating social media for information-gathering, considering the high probability of misinformation
Ambelu et al [33], 2021	Facebook, Twitter, Zoom, etc	Those receiving information from social media have significantly higher odds of experiencing psychological distress	Misinformation and myths about the COVID-19 pandemic bombarding social media, which strengthened groundless stress about COVID-19 among the population	Developing an intervention plan to intervene in the psychological distress in the population, mainly targeting those groups who received information from social media
Wagner, and Reifegerste [34], 2021	Facebook, Twitter, Zoom, Facetime, etc	Misinformation on COVID-19 influencing others and providing misleading orientation	Information-seeking and orientation-seeking practices in and through communication via social media	Examining people’s information-seeking and orientation-seeking practices in and through communication about pandemic-related media coverage could help us judge the importance of (constructive) media coverage and, ultimately, contribute to understanding the processes hindering and fostering public health compliance

#### Types of Social Media

A total of 33% (3/9) of studies [3,27,28] did not mention specific social media forms contributing to COVID-19–related health inequalities: internet [3], social media [28], and digital media [27]. The remaining 67% (6/9) of studies referred to concrete social media used: Sina Weibo [29]; Twitter [30,31]; Twitter, YouTube, and Google engine [32]; and Facebook, Twitter, Zoom, etc [33,34].

#### Types of Social Media Use–Related Health Inequalities and Associated Factors

Overall, 4 broad types of social media use–induced health inequalities were revealed in the 9 studies: disparities in the access to COVID-19–related health information [3,27-29]; misinformation regarding COVID-19 and associated precautions [29-32,34]; Twitter accounts receiving disproportionate attention for COVID-19 content during the pandemic [30]; and those obtaining information from social media having significantly higher odds of experiencing psychological distress [33].

In the study by Wang et al [3], in the context of traditional media (eg, newspapers, radio, and television) failing to improve knowledge levels, people with different educational backgrounds acquired different amounts of COVID-19–related health knowledge through the use of the internet, leading to health inequalities during the COVID-19 pandemic. Such COVID-19–related health inequalities created the digital health knowledge gap between individuals with diverse education levels, which influenced them differently in terms of COVID-19–related health behaviors and medical decisions. In contrast, 22% (2/9) of studies [27,28] did not consider the study participants’ education but rather dealt with the COVID-19–related health inequalities between people with disabilities and people without disabilities. A study by Dai and Hu [27] described disabled people’s inadequate, accessible information about COVID-19 in comparison with nondisabled people owing to the gaps between policies and practices regarding the digital accessibility infrastructure. Specifically, the accessible information on the web designed for people with disabilities focused little on applicable information to meet their needs during the pandemic; official press conferences and television news on COVID-19 failed to use sign language interpreters to inform people with disabilities of the latest situations of the pandemic. There was no accessibility design on the newly developed website for disseminating information on COVID-19, making it inaccessible to people with hearing or visual disabilities. The study by Almusawi et al [28] was similar to the study by Dai and Hu [27]. Similar to the study by Dai and Hu [27], the study by Almusawi et al [28] was also concerned with people with disabilities, specifically participants with hearing loss. However, unlike the study by Dai and Hu [27], the study by Almusawi et al [28] focused on the reliance on different information sources: people with hearing loss mainly relied on social media while people with no hearing loss mainly relied on official government sources. Besides, unlike studies by Wang et al [3] and Dai and Hu [27], the study by Almusawi et al [28] touched upon the hearing participants’ low health literacy, which prevented them from accessing health information on the web.

Misinformation was the most prevalent topic in the 9 selected studies. Of these, 56% (5/9) of studies [29-32,34] dealt with COVID-19 misinformation on various social media platforms and related precautions. The study by Zeng and Li [29] discussed disparities between East and Central China and West China in health information and misinformation released on a popular social medium in China named Sina Weibo. The contributors were government public health authorities’ inadequate willingness and ability to use government Weibo accounts, their inactive operation of social media, and the Weibo accounts’ failure to respond to public inquiries and huge amounts of COVID-19–related misinformation promptly. In contrast, 4 studies [30-32,34] investigated COVID-19–related misinformation on Twitter, Facebook, Zoom, YouTube, Google, Facetime, etc, which led to negative outcomes of COVID-19 prevention and treatment, psychological problems, and misleading orientation. The underlying factors included purposeful or purposeless amplification of COVID-19 information, people’s preference for social media over government organs, and people’s orientation-seeking.

#### Proposed Resolutions to Health Inequalities

Table 3 shows that although the proposed resolutions to COVID-19–related health inequalities are mostly specific to each of the 9 selected studies, what they have in common is intervention on the part of different players including government public health authorities [3,27-30,33], university authorities [32], and scientific communities [31,34]. The intervention measures are concerned with the establishment of relevant platforms [3,27], the development of related programs [3,32,33], the improvement of communication strategies [3,27-30], and the investigation of information-seeking, information application, and information orientation practices [31,34].

## Discussion

### Principal Findings and Implications

The findings on COVID-19–related health inequalities induced by the use of social media and recommended resolutions reported in the 9 studies were classified into 4 categories and discussed in the following subsections. Meanwhile, the relevant implications of each category were discussed.

#### Health Information Inaccessibility–Induced Health Inequalities and Proposed Resolutions

With advances in new media and IT, it is of great value to study the COVID-19–related knowledge gap and digital technology–induced unequal health information distribution [3], which were caused by the “Digital Divide” [36], that is, the gaps in the access to and use of the internet among different social groups leading to knowledge gaps [36]. Access gaps may not necessarily breed COVID-19 knowledge gaps because people use the internet media as the most frequent and dependent way to acquire COVID-19–related information [3]. In this case, what induced COVID-19–related health inequalities was use gaps: disparities in intensity, behavior, content, literacy, and other aspects when using the internet media [37,38]. To address the health inequalities caused by the access and use gaps of social media, the following resolutions were proposed: (1) improving the pertinence in the ways and contents of social media–based communication; (2) building social media platforms for authoritative scientific COVID-19 knowledge communication; and (3) developing social media literacy and science literacy of the public [3].

Compared with the people with no disabilities, people with disabilities faced more barriers when accessing health information during the COVID-19 pandemic for two main reasons: (1) the government’s commitment to information accessibility was not always fulfilled, leading to the neglect of the needs for information in people with disabilities; and (2) the newly established web sites for COVID-19 information dissemination lacked accessibility design, thereby being inaccessible to people with disabilities, especially the people with hearing or visual disabilities [27,28]. To eliminate these health inequalities, Dai and Hu [27] proposed a self-initiated and volunteer-driven Disability Support Network, which fully considered various dimensions of information in terms of formats, content, and situations for people with disabilities, and government authorities and public service sectors taking more proactive steps to provide inclusive communications and information in emergencies for people with disabilities on social media. Besides, it is necessary to bridge the gap in health literacy for people with hearing loss using social media and web-based unstandardized dialectic writing and adopting different ways of disseminating information linking to web-based videos in sign language, to ensure equal access to health care and universal compliance with health directives at the population level [28].

The use of social media by public health authorities (eg, Center for Disease Control and Prevention) helped popularize daily health information through Weibo accounts, especially during COVID-19 [29]. However, the high dropout rates of Weibo accounts in some areas and the unequal distribution of Weibo accounts between the eastern region and the middle and western regions caused health inequalities among people in terms of access to helpful information on epidemic prevention and control [29]. The passives state and low willingness and ability of social media operations of Chinese public health authorities and their failure to respond to public inquiries and large amounts of misinformation on social media during the epidemic promptly made misinformation on social media even more rampant, causing even greater health inequalities. Governments in the central and western regions need to learn from the similar experiences of governments in the eastern region, maintain their social media activity by updating daily information frequently, and use social media to release public health information to the public promptly [29].

#### Misinformation-Induced Health Inequalities and Proposed Resolutions

The role of social media in breeding misinformation attracted the attention and aroused the concern of Wang et al [3]. Social media make it very easy for misinformation and fake news about COVID-19 to spread to the public [31]. A good case is a misinformation on Hydroxychloroquine on Twitter. Interestingly, Donald Trump and his supporters turned out to be the most influential actors in advocating hydroxychloroquine as an effective treatment for coronavirus on Twitter [31]. People’s trust in social media sources over government organs (eg, Center for Disease Control and Prevention and World Health Organization) [32] and their preferred information-seeking and orientation-seeking practices via social media [34] made misinformation on social media platforms even more unconstrained. Misinformation regarding the pandemic frequently appears on social media platforms, serving as a source of health risk [39,40].

Therefore, the media outlet should be more responsible for monitoring health message dissemination [41]. An effective way of countering misinformation lies in the gatekeeping of incorrect information, which helped to fight against the spreading of misinformation during COVID-19 owing to its ability to mediate and fact-check the accuracy of the contents [42]. Another way is to use the disproportionate voice of the crowdsourced elites on the web because people crowdsourced a small set of accounts on social media when clear information about COVID-19 prevention and protection was missing [30]. To mitigate misinformation in college students, university authorities need to consider carrying out programs to aid them in navigating social media for information-seeking and considering the high probability of misinformation [32].

“As networked societies become more accustomed to relying on information from varying sources on social media outlets and other cyberspaces (even for critical medical knowledge), the implications of how they interpret and apply that information in physical spaces is a significant consideration” [31]. Therefore, investigating people’s information- and orientation-seeking practices in and through social media–based communication about COVID-19–related social media coverage [34] can help find practical approaches to minimize misinformation on social media, which most possibly caused health inequalities during the pandemic. What is needed to combat COVID-19–related misinformation on social media is (1) a keen sense of responsibility and the capability to think critically before sharing any information regarding the SARS-CoV-2 virus on social media platforms [31] and (2) a rational judgment of which social media sites are trustworthy and the ability to read and interpret health information on social media critically [32].

#### Disproportionate Attention to COVID-19 Information and Proposed Resolutions

Gallagher et al [30] studied the Twitter accounts receiving disproportionate attention during the COVID-19 crisis and the variation across demographics, finding that the public crowdsourced journalists, media outlets, and politicians more than epidemiologists, public health officials, and medical professionals. COVID-19–related health inequalities may arise owing to (1) crowdsourced elites varying across demographics in terms of race, geography, and political alignment; (2) different subpopulations preferentially amplifying elites who are demographically similar to them; and (3) different subpopulations crowdsourcing different elite accounts (eg, journalists, elected officials, and medical professionals) in different proportions [30]. Paradoxically, by working with COVID-19 elites, epidemiologists, public health officials, and medical professionals to popularize scientifically informed health guidelines and debunk misinformation, it is most likely to leverage the crowdsourcing potential of social media to achieve more health equality [30].

#### Higher Odds of Social Media–Induced Psychological Distress and Proposed Resolutions

In the context of a severe public health emergency, the public depends heavily on media coverage to stay informed [34]. COVID-19 has bred a massive “infodemic” [43] where various social media bombarded people with misinformation and myths about the COVID-19 pandemic, which intensified their groundless anxiety and stress about COVID-19 [33]. As social media exposure was associated with anxiety [44], it is necessary to develop an intervention plan to intervene in people’s psychological distress, especially targeting those who predominantly received COVID-19–related health information on social media platforms [33]. People, especially those experiencing greater psychological distress, need to exercise extreme caution when deriving information on COVID-19 from social media and better use information delivered by the World Health Organization’s “infodemics” team [45]. Moreover, they are encouraged to communicate with others about social media coverage of COVID-19 health information to understand better and evaluate pandemic-related information [34].

### Limitations

This systematic review has some limitations. First, 2 databases, Embase and CINAHL, were not used for retrieving relevant studies owing to our inaccessibility to these databases, possibly making some related studies unidentified from the literature. This is to the detriment of the comprehensive synthesis of the principal findings reported in extant studies. Besides, some principal findings were likely to have low generalizability, considering that some social media use–induced health inequalities and the associated factors and recommended resolutions were reported in only one selected article. Moreover, we failed to compare the principal findings of this review with other systematic reviews, for this review was the first one concerning this topic. Finally, there was no protocol for how to report social media use–induced health inequalities when this review was performed, so certain reporting biases may be involved in this review. Future research will benefit from developing a reporting protocol for evaluating studies on social media use–induced health inequalities based on current frameworks.

### Conclusions

This was the first systematic review seeking (1) to identify and summarize COVID-19–related health inequalities induced by social media and the associated contributing factors and (2) to characterize the relationship between the use of social media and health disparities during the COVID-19 pandemic. The findings synthesized from the selected studies highlighted the great value of studying the COVID-19–related knowledge gap and the digital technology–induced unequal health information distribution and the resulting health inequalities, providing knowledge about the relationship between social media use and health inequalities regarding health knowledge and precautions against COVID-19. The 4 categories of COVID-19–related health inequalities induced by the use of social media and the associated contributors and recommended resolutions summarized in this review can provide some empirical evidence for developing practical solutions to help solve the health inequalities caused by social media use in the context of the repeated resurgences of the pandemic and future public health emergencies and crises. Informed by this review, researchers, social media and health practitioners, and policy makers can join hands to take full advantage of social media to promote health equality while minimizing social media use–induced health inequalities [21].

## Abbreviations

PRISMA: Preferred Reporting Items for Systematic Reviews and Meta-Analyses
